# Dual-Stream Architecture Enhanced by Soft-Attention Mechanism for Plant Species Classification

**DOI:** 10.3390/plants13182655

**Published:** 2024-09-22

**Authors:** Imran Ullah Khan, Haseeb Ali Khan, Jong Weon Lee

**Affiliations:** Mixed Reality and Interaction Laboratory, Department of Software, Sejong University, Seoul 05006, Republic of Korea; imrankhan@sju.ac.kr (I.U.K.); haseebalikhan@sju.ac.kr (H.A.K.)

**Keywords:** plant species classification, deep learning, dual-stream architecture, dilated convolution, soft attention

## Abstract

Plants play a vital role in numerous domains, including medicine, agriculture, and environmental balance. Furthermore, they contribute to the production of oxygen and the retention of carbon dioxide, both of which are necessary for living beings. Numerous researchers have conducted thorough research in the classification of plant species where certain studies have focused on limited numbers of classes, while others have employed conventional machine-learning and deep-learning models to classify them. To address these limitations, this paper introduces a novel dual-stream neural architecture embedded with a soft-attention mechanism specifically developed for accurately classifying plant species. The proposed model utilizes residual and inception blocks enhanced with dilated convolutional layers for acquiring both local and global information. Following the extraction of features, both streams are combined, and a soft-attention technique is used to improve the distinct characteristics. The efficacy of the model is shown via extensive experimentation on varied datasets, including several plant species. Moreover, we have contributed a novel dataset that comprises 48 classes of different plant species. The results demonstrate a higher level of performance when compared to current models, emphasizing the capability of the dual-stream design in improving accuracy and model generalization. The integration of a dual-stream architecture, dilated convolutions, and soft attention provides a strong and reliable foundation for the botanical community, supporting advancement in the field of plant species classification.

## 1. Introduction

Plants play a crucial part in people’s lives for a variety of reasons, including growing concerns about a deficiency of nutritious food around the globe with an exponentially growing population and changing climates globally [1]. Modern plant agriculture techniques are enormous because they have a substantial impact on the national economy and personal aspects of people’s lives [2]. Plant species are useful for medicine, food, and industrial applications. They also play a vital role in a country’s economic growth. Environmentalists are also interested in the automation of plant identification by developing a system based on spatiotemporal characteristics extracted from their structure and appearance. Conventional plant species recognition methods are costly and time-consuming because they require the manual involvement of human specialists [3]. Some plants are on the verge of extinction. As a result, it is critical to establish a database for plant protection. The most basic or traditional procedure is the naked-eye examination of the plant, and this procedure entails constant expert observation of a variety of farm areas [4,5], which is a lengthy and costly procedure. The automatic classification of the plant species is key to achieving plant protection and safety and making the process easy for classifying the plant species [6]. Plant leaves are generally the most prominent parts for identifying species, as opposed to flowers, which are only available for a limited time. As a result, the leaves are an optimal choice for developing a system for the automation of plant recognition. The leaves play an important role in understanding plant genetic relationships, structure, and plant development. Plant identification is a difficult task, including for botanists, due to the existence of a large number of species in the world [7,8]. Botanists use leaf recognition technology to classify the species of various plants. Plants have distinct characteristics that vary in many ways, such as size, texture, color, and shape, and they are different due to these characteristics [9]. There are several “Computer-Aided Detection” (CAD) techniques that have been used for plant recognition based on their leaves, where in recent years, [10,11] achieved significant classification accuracy in plant species recognition.

Machine-learning and deep-learning approaches have become more popular for classifying plant species, providing potential opportunities for automating plant species identification [12]. Several researchers have investigated the use of different machine-learning algorithms and deep-learning architectures for this problem, including both conventional classifiers and advanced neural networks [13,14]. Although these systems failed to obtain high levels of accuracy, there is a prevailing tendency to choose simpler structures since they are easier to execute and deploy to the edge devices. Fundamental designs often consist of shallow neural networks or traditional machine-learning methods like Support Vector Machines (SVMs) or Random Forests. Although these approaches may provide acceptable outcomes in certain circumstances, they often demonstrate constraints when confronted with complicated datasets or a wide range of plant species. Deep-learning models, which are renowned for their capacity to acquire intricate hierarchical representations from unprocessed data, have gained significant popularity in the field of plant species categorization [12]. Nevertheless, several deep-learning models used in this particular domain encounter drawbacks such as overfitting, computational complexity, and lack of interpretability. Despite these difficulties, researchers persist in investigating innovative methods and structures to surmount these constraints and improve the precision and resilience of plant species classification.

Plant species recognition and classification accuracy are essential in many fields, including environmental protection and agriculture. Making use of recent developments in deep learning, a unique model architecture has been created explicitly for the accurate visual cue-based recognition of plant species. Our proposed cutting-edge model combines a two-stream architecture, using RGB image data for both streams as an input to enhance its discriminative power. Dilated Convolutional Neural Network layers (DiCNN) are utilized in the first streams to analyze RGB images and extract complex spatial hierarchies and patterns. In addition, a second stream processes the same data and acquires different types of patterns and features from the input data. Additionally, a soft-attention mechanism is employed in the model, which focuses on the most prominent features and ignores the less prominent features. This attention module enhanced the model’s capability to deal with complex images and made it more expert in identifying the different plant species. By using the attention process and encapsulating the dual-stream integration, our proposed model has the potential to correctly classify the plant species from their visual structures. This all-encompassing architecture, which combines several dilated convolutional, pooling, residual, and inception layers, creates a strong foundation for classifying plant species. It uses a variety of techniques to identify complex features that are necessary for the precise identification of species. The proposed technique presents the following contribution.

1. We contribute a novel dataset of indoor plant species, which comprises 48 different classes. This dataset is a valuable resource for further progressing plant species recognition with diverse classes, and it allows researchers to further develop more effective techniques in the field of plant species classification.
2. We developed a dual-stream network that utilizes two parallel streams, such as the residual block and inception block, embedded with dilated convolutional layers to extract more prominent features from both streams. In contrast to existing pre-trained models, that used shallow architecture, our proposed model used two parallel streams to enhance the feature-extraction process across different plant species for accurate classification.
3. Our proposed dual-stream model is further strengthened by using a soft-attention mechanism. The integration of a soft-attention mechanism enhances the model’s adaptability to focus on more prominent features by dynamically amplifying and weighting salient data from both streams while ignoring the irrelevant features.
4. We performed a detailed ablation study and comparative analysis of the proposed model with other baseline models. The results indicate that the proposed model outperforms all the other baseline models in terms of accuracy, precision, recall, and F1 score.

The subsequent sections of the article are structured as follows: Section 2 discusses the theoretical background, major trends, and substantial drawbacks associated with existing algorithms. Section 3 provides a detailed overview of each module employed in the proposed network. Additionally, Section 4 includes the experimental setting, dataset description, and comparative analysis in terms of quantitative and qualitative against various competitive benchmarks, followed by a detailed ablation study that represents the effect of each module used in the proposed method. Finally, we conclude the article and provide the future direction of the research in Section 5.

## 2. Literature Review

In recent times, computer vision research has found interest in the study of plant classification through image processing [15]. The organs of plants, such as their leaves, roots, fruits, flowers, skins, stems, and seeds, can be used to categorize and identify different species of plants. Compared to other organs, leaves are essentially in a plane state and have a stable shape and structure, which is advantageous for two-dimensional image processing. Leaves are abundant and easily harvested throughout practically all growth seasons, whereas roots are inaccessible and flowers are only available during flowering seasons. That is the reason plant species classification systems most typically depend on leaves. Since leaf-based plant species recognition does not require the expert knowledge of a botanist, it is preferred over molecular biology techniques [16,17]. Plant species can be identified by the form, color, texture, and other properties of their leaves. However, the leaf color trait is rarely utilized to identify plant species as leaf colors change with the seasons and the environment. In nature, the leaf-form properties are relatively stable. In morphology, the diversity of leaf forms is a crucial foundation for plant classification and recognition [18].

Researchers have recently combined neural networks and computation devices to address many problems in this modern world, specifically in providing assistance to human beings. They have indicated that image-based assessment systems offer more accurate and consistent findings than human visual inspections [19] in plant species classification and plant disease detection [20,21]. Accurate classification of medicinal plants is vital for individuals involved in the preparation of Ayurvedic medicine, botanists, forest department offices, farmers, and practitioners. There has been plenty of effort into classifying objects using various methodologies, where [22] established CNN as a fundamental deep-learning tool for introducing deep-learning model strategies in classification and detection. Deep-learning models have been employed in agriculture to a limited level in recent years. One kind of dynamic model that can be useful for classification applications is CNNs. There are numerous CNN models for classification, including GoogLeNet [23], AlexNet [24], ResNet50, ResNet18, ResNet101 [25], DenseNet [26], VGG16, VGG19 [27], SqueezeNet [28], and various others. Using AlexNet and GoogLeNet, Mohanty et al. [29] were able to classify 14 distinct plant leaves with an accuracy of 99.27% and 99.34%. The authors have used a variety of input data, including color images, segmented images, and grayscale images. Another research [30] used a CNN model to classify plant leaf data and obtained 86.2% accuracy. Some studies focused on developing comprehensive systems for plant classification, wherein various machine-learning methods and feature-extraction techniques were employed [31]. These studies collectively illustrated the efficacy of integrating advanced imaging techniques with machine-learning technologies for plant species classification, which remained a critical aspect of research in plant science and phytotechnology. Likewise, the researchers in [32] presented CNN-based plant classification (CNN-PC) models for efficient plant species identification. The model generated pre-trained weights based on input plant image data and handled dependencies. The model’s performance was evaluated using the Vietnam Plant (VNP-200) dataset, which achieved a classification accuracy of 96.42% across all 200 classes. Moreover, the authors in [33] proposed a novel feature-extraction method using semi-supervised learning techniques and masked autoencoder architecture, MAE SGD, which achieved nearly 94% accuracy in classifying 710 plant species on the QuangNamForestPlant dataset. In the efforts to classify plant leaves with high accuracy, Barre et al. [34] used the LeafSnap, Foliage, and Flavia datasets for the classification of different classes using their suggested model LeafNet, where 60 classes from the Foliage dataset and 184 classes from LeafSnap were classified with an accuracy of 95.8% and 86.3%, respectively, while on 32 classes in the Flavia dataset, they obtained 97.9% accuracy. A deep CNN model in [35] used the “Multilayer Perceptron” (MLP) classifier, which achieved an accuracy of 97.7% on 44 classes in the MalayaKew dataset, and this accuracy was increased to 98.1% with the SVM classifier. Additionally, a study [36] conducted a plant classification task that utilized geometric characteristics in preliminary processing. They presented the 3SN Siamese network, which gained knowledge for the leaf classification task from both spatial and structural characteristics. It successfully classified 10 plant species from the Fla-via dataset with an accuracy of 90%. Gao et al. [37] completed a LifeCLEF Plant Identification task with an accuracy of 84.2%. Similarly, Zhu et al. [38] used a two-way-attention CNN model to recognize plant families and further identify plant classes for the four different datasets. In [39], the accuracy of classifying medicinal plants using the AlexNet model was 94.87%, while the Ayurleaf CNN model achieved 95.06% accuracy for 20 species of self-collected medicinal plant data.

Duong-Trung et al. [40] used the MobileNet model to achieve 98.5% classification accuracy. Liu et al. [41] developed a 10-layer CNN model for plant leaf classification and obtained an accuracy of 87.92% across 32 classes. With the LeafSnap dataset, the researchers in [11] implemented a ResNet model that achieved a classification accuracy of 93.09%. The authors in [7] completed plant leaf classification on images taken by [42] with the help of an Apple iPad device where the Deep Neural Network (DNN) model achieved an accuracy of 91.17%, while the CNN model has 95.58% accuracy. Using photos taken with mobile devices, Yang et al. [8] performed leaf classification of various plants with complicated backgrounds. The classification accuracy for the Inception ResNetV2 model, VGG16, and VGG 19 model was 89.6%, 91.5%, and 92.4%, respectively. Additionally, a study [43] that employed the AlexNet model to identify berry plants was able to achieve 97.80% accuracy for the three classes of data that the researchers collected themselves. The authors of [44] presented a novel method for classifying plants that combined key point identification with SIFT, morphological transformations, and leaf edge features. With a 95.62% accuracy rate on the PlantVillage dataset, the method outperformed conventional techniques that classified plant species based only on leaf shape and texture. Moreover, the importance of deep learning is demonstrated by a study [45] that used transfer learning with VGG-19 for plant recognition in Swedish leaves and achieved an amazing accuracy of 99.70% in identifying 15 different tree classes. Furthermore, using leaflet features to classify plant species, the paper [46] presented the Conv2D Xception architecture with the Adadelta Gradient Descent (CXAGD) deep-learning model. Compared to other CNN models, it achieved high accuracy (97.85%), precision (97.42%), recall (97.75%), and F-score (97.76%). Using the CXAGD, the model was trained on a dataset of 4500 vegetable leaflets. Using Canny edge detection and VGG16 on the Flavia dataset with 15 classes, the authors of [47] used leaf vein patterns to identify plant species with 95% accuracy. The approach highlighted how crucial it is to identify unique qualitative traits from leaf pictures to accurately classify plants. A concise description of various benchmarks over distinct datasets, along with their techniques, is shown in Table 1. In addition, the general limitations associated with the existing techniques are provided in the following points:

- The existing techniques often struggle with generalization to diverse environmental conditions and class imbalances, necessitating more robust algorithms.
- Various existing algorithms of both machine learning and deep learning require significant computational resources for training and deployment, posing challenges for resource-constrained environments.
- The publicly available datasets contain diversity and class imbalance problems, which hinder the model training and generalization, highlighting the need for improved dataset curation and augmentation approaches.

## 3. Proposed Methodology

This section explains the proposed methodology of our technique, where we have explained the details of our proposed dual-stream architecture embedded with a soft-attention mechanism. We performed an ablation study that involved various methods to improve our model accuracy in terms of plant species classification. Firstly, we have used a hybrid model with different residual and inception blocks for classification. Secondly, we used these blocks, followed by an attention mechanism, which improves the model performance. An attention mechanism is a fundamental neural network approach for classification tasks that allows neural networks to selectively highlight pertinent features or portions of the input data. Thirdly, we have used the residual block and inspection block in the dual stream, where the residual block is in a separate stream and the inception block is used in a separate stream for the extraction of pertinent features from both streams. Lastly, we proposed our more accurate and efficient model, where we use a dual-stream network of residual block and inception block and then concatenate them and pass them to the soft-attention mechanism. Adding soft-attention mechanisms enhances the neural network’s ability to recognize and utilize important information, which results in increased classification accuracy and resilience over a variety of datasets and classes.

### 3.1. Proposed Dual-Stream Approach

Our proposed approach presents a novel dual-stream architecture that utilizes the two parallel blocks enhanced with dilated convolutional layers, which is skilled in capturing the most prominent features from the input data. By using the shortcut connections, residual blocks facilitate feature learning and gradient flow efficiently, and these connections also help in vanishing gradient problems, which is common in deep-learning models. The second stream, which comprises an inception block, is utilized to capture multiscale features and the most complex features from the data through receptive parallel convolutional routes by using receptive fields. Two input tensors, which represent the residual and inception block routes, respectively, are used at the beginning of the algorithm. Each stream’s ensuing dilated convolutional and pooling layers are customized for the particular block type. For example, the first stream includes inception block with several parallel dilated convolutions for feature diversification, whereas the second stream includes residual block with unique shortcut connections and dilated convolutional processes. Incorporating dilated (or atrous) convolutional layers into the residual block and inception block improves the receptive field and collects contextual information. A typical residual block comprises a shortcut connection, also known as a skip connection, and two convolutional layers. Dilated convolutions can substitute or enhance the conventional convolutional layers in the residual block. The inclusion of dilated convolutions inside the residual block allows the network to effectively capture a broader context or global information while keeping the number of parameters relatively the same. By augmenting the dilation rate inside the designated residual block, the model concentrates on gathering information over a wider spectrum. Hence, it enhances its effectiveness for tasks necessitating extensive interdependencies. Let’s suppose X represents the input, $F\left(X\right)$ identifies the shortcut connection, $Conv\left(X,W,\;dilation\;rate\right)$ shows the dilated convolution layers, and Y represents the output. Therefore, Equation (1) can be written as:

(1) $$Y=F\left(X\right)+Conv\left(X,\;W,\;dilation\;rate\right),$$

On the other hand, the inception block is composed of many concurrent branches, each applying a distinct processing method to the input. Typically, these branches consist of convolutional layers that have variable kernel sizes. Incorporating dilated convolutions into one of the branches of the inception block allows for a broader context to be considered during the process of feature extraction. Let’s suppose X is the input, $Concat\left(Conv_{1\times 1}\left(X\right),\;Conv_{3\times 3}\left(X\right),Conv_{dilated}\left(X,dilation\;rate\right),\ldots \right)$ represents the different convolutional branches and dilated convolutional layers within the inception block, and Y is the output. So, mathematically, it can be expressed in Equation (2) as:

(2) $$Y=Concat\left(Conv_{1\times 1}\left(X\right),\;Conv_{3\times 3}\left(X\right),Conv_{dilated}\left(X,dilation\;rate\right),\ldots \right)$$

The network’s capability to collect characteristics at several scales enables it to effectively identify patterns of different dimensions. Dilated convolutional layers have an impact on both residual and inception blocks by allowing the network to gather contextual information from a wider region without adding much computational burden. It is of utmost importance for tasks that need the comprehension of both local and global features, such as image recognition. The use of dilated convolutions in these blocks varies depending on the architecture and the task’s unique demands.

**Figure 1:**
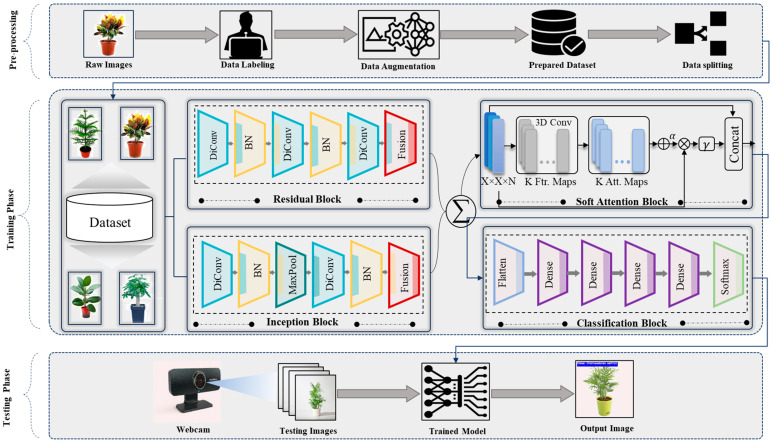
A high-level generic diagram of the proposed study, comprising three distinct phases: Phase (1) contains different augmentation techniques to mitigate the data-scarcity problem. Phase (2) showcases a dual-stream network, employing two different networks, such as residual block and inception block, followed by a soft-attention module for effective feature learning. Finally, Phase (3) signifies the testing phase, where the proposed network is evaluated on testing data.

Following the separate processing in both streams, a concatenation layer is used to combine the results to incorporate the unique features that each pathway has acquired. A technique for adaptively emphasizing significant characteristics from the concatenated representations is then presented. The soft-attention mechanism is used to enhance the model architecture, which comprises a pooling layer, dense layer, and reshaping, and the features are further refined by multiplication between attention weights and concatenated features, which improves the concentration of the proposed model on more prominent features [48,49]. The architecture mainly focuses on the separation and fusion of feature-extraction algorithms through the use of inception and residual blocks in parallel streams. Combining these disparate representations allows for a more thorough exploration of feature space, which is further improved by a soft-attention mechanism to allow for adaptive feature refining. To accommodate complex and varied feature representations for plant species classification tasks, the suggested architecture aims to maximize the special abilities of both residual and inception blocks. The principal objective is to increase classification performance in terms of accuracy and resilience. Figure 1 shows the general architecture of the proposed framework.

### 3.2. Soft-Attention Mechanism

**Figure 2:**
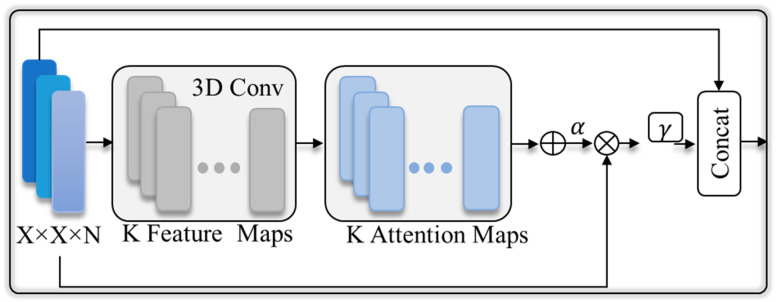
The soft-attention function of converting feature map to attention maps and postprocessing procedure.

Soft-attention mechanisms are crucial for improving the capabilities of neural networks as they allow the networks to selectively concentrate on particular areas of the input image. Within the realm of image classification, these methods are very important for identifying pertinent characteristics and patterns within an image. Our suggested architecture utilizes the soft-attention mechanism, which is applied strategically after combining the characteristics from the dual-stream residual block and the inception block. The soft-attention mechanism operates by allocating attention weights to distinct regions or channels of the input feature maps. Although our dual-stream architecture has the ability to extract features from the images using parallel blocks, it is not effective in each situation like in low lighting, which can affect the model’s capability of correctly classifying the plant species in indoor scenarios. Therefore, we utilized a soft-attention module, which effectively extracts the most pertinent features from the input images. The attention mechanism we utilized in this task consists of the PReLU activation function, max pooling layer, soft-attention unit, concatenation, and dropout layer in the last. The activation function assesses the input from the preceding layer and transmits its weights instantly to the soft-attention unit and pooling layer. The feature tensor $\left(b\right)$ is used as the input in the soft-attention unit of the deep-learning model.

(3) $$f_{sam}=ab\left(\sum_{k=1}^{K}softmax\;\left(W_{k}.b\right)\right),$$

where the input of the 3D convolutional layer is $b\in \;R^{h\times w\times d}$, the weights are represented by $W_{k}\in \;R^{h\times w\times d\times N}$, and the 3D weights are denoted by N. The convolution result is normalized via the SoftMax function to produce K attention maps where *K* = 16. The attention maps are merged to create a single attention map that functions as a weighting factor α, as seen in Figure 2. Later, the variable α is multiplied by t to precisely transform the values for essential features, and a is utilized for further scaling since particular images need different a values, with a considered as a training parameter. A residual branch is created by combining the precisely scaled features $f_{sam}$ from Equation (3) with the original features b. While training our model, the value of y is set to 0.01, which allows the network to progressively adjust its attention needs and transmit the weights to maximize pooling layer 1. The pooling layer 2 acquired the core features directly from the PReLU activation function. Pooling layer 1 and pooling layer 2 feature maps are combined and then inputted into the PReLU activation functions, followed by a dropout layer. The soft-attention block assists the network in prioritizing the plant that is far from the camera or under low light conditions. The dual-stream architecture, with the included soft-attention technique, is well-suited for the classification of indoor plant species.

## 4. Experimental Results

This section offers a concise overview of the experimental setup, followed by a detailed explanation of the datasets utilized for the experimental analysis. Additionally, we provide an in-depth exploration of both quantitative and qualitative analysis in terms of comparing the performance of the proposed network with other state-of-the-art (SOTA) techniques. For the experimental results, we implemented the proposed network on intel corei9 CPU with Nvidia GeForce 3070 GPU (Nvidia Corporation, Santa Clara, CA, USA) embedded with 8 GB memory. Additionally, we used a well-known framework named Keras as the front end and TensorFlow was utilized as the back end. Further, we reimplemented various competitive techniques on both datasets and trained them for 100 epochs with a batch size of 32, as well as SGD optimizer with 0.0001 learning rate and weight decay while using a 0.3 dropout. We also used early-stopping mechanisms on the basis of validation loss and to prevent the model from overfitting. All these optimal values for regularization techniques and hyperparameter tuning are selected after performing different experiments with different ranges of values. We performed many experiments by changing the values of dropout rate, weight decay, learning rate, and early-stopping mechanism to select the optimal values at which the model provides its best performance and accuracy. For the model evaluation, we used accuracy, precision, recall, and F1 score, which are very common evaluation metrics in image classification tasks. The purpose of this experimental study is to obtain a deeper understanding and comparison of proposed model with other baseline models. The ablation study examines how different elements collectively influence the performance, reliability, and interpretability of the model in plant species classification.

### 4.1. Dataset Description and Pre-Processing

This section provides a comprehensive description of the two datasets utilized in our experimental analysis to determine the efficacy of the proposed network against various competitive techniques. Initially, we conducted extensive experiments over Flower-299 dataset, which contains 299 different species. A detailed description of those species is available in [50]. This dataset composes 115,944 different images, with an average width of 271 pixels and height of 242 pixels. The images are distributed into different classes, where the average number of samples per label is 387 images, as detailed in Table 2. This variation in the number of images per class reflects the diversity of different classes within the dataset. Such diversity is essential for robust training of deep-learning models, allowing the model to generalize well across all classes. The Flower-299 dataset is considered to be widely used in the plant domain. However, the dataset contains several limitations that need to be addressed. One notable limitation is the potential class imbalance, where certain flower species may have significantly fewer images compared to others. This imbalance can lead to biased model training and reduced performance, especially for underrepresented classes. Additionally, the dataset may lack diversity in terms of environmental conditions, such as variations in lighting, background, and image quality. Such uniformity may hinder the model’s ability to generalize well to real-world scenarios where environmental conditions can vary widely.

**Figure 3:**
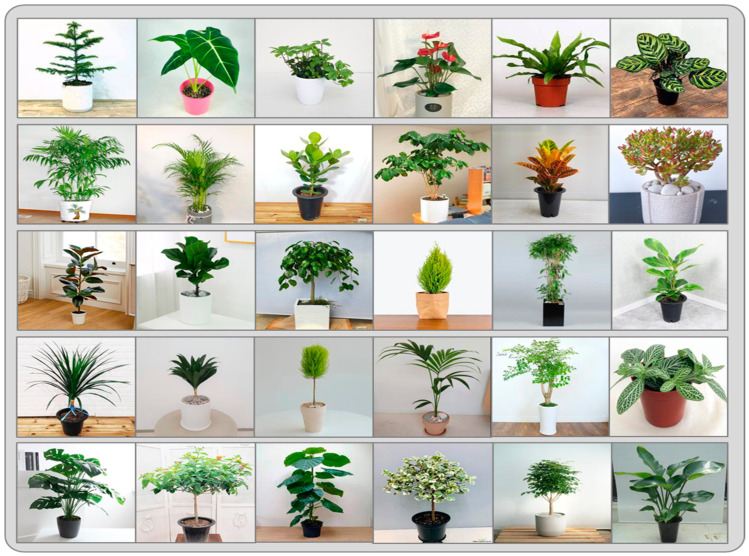
Shows the different types of plant species samples from our proposed dataset.

To address these limitations and ensure robust model training and performance, we developed our custom dataset, which comprises 48 distinct categories of plant species. The first version of the data was acquired from a plant firm using a “Canon 450D + EFS 18–55mm” camera (Canon Oita Factory, Oita, Japan), while the details about the dataset samples are mentioned in Table 2. Figure 3 shows the samples taken from our proposed plant dataset. The plants were cultivated inside, and the images were captured using two 45W flexible LED lamps. The images were captured from a distance of roughly 120 cm, using angles of 0 degrees, 45 degrees, and 60 degrees. To standardize the picture formats, we converted all the acquired photographs to .jpg format during the pre-processing step. We modified the dimensions of all the images to 3 where the dimension refers to the measurements of the length and width of an image. The measurement is often conducted in pixels, and we ensure that each image has a consistent depth of 24 bits. Following this step, we apply some more pre-processing steps involving different augmentation techniques to increase the number of samples because the deep-learning model needs to be trained on a huge variety of samples so that it classifies each plant species accurately. We used the Albumentations library to create 10 separate augmentation pipelines. Each pipeline is designed to add various variations and perturbations into input images hence enhancing the dataset for training deep-learning models. The augmentation techniques used in these pipelines encompass several transformations designed to improve the resilience and flexibility of the model. Operations such as random rotations, flips, variations in brightness contrast, and the use of Contrast-Limited Adaptive Histogram Equalization (CLAHE) enhance the capacity to adapt to different lighting situations. In addition, this dataset includes blur effects, gamma changes, and simulated weather conditions such as rain, fog, sun flare, and shadows to enhance its diversity and robustness. 

### 4.2. Evaluation Metrics

The study utilized several evaluation indicators to determine the robustness of the proposed network and various SOTA techniques. These indicators include Precision, Precision, F1-Score, and Accuracy, as the detailed descriptions are mentioned in the following references [51]. These evaluation metrics are calculated through True Positive (TP), True Negative (TN), False Positive (FP), and False Negative (FN). The mathematical formulation of evaluation metrics is mentioned in the following equations:

(4) $$Accuracy=\frac{TP+TN}{TP+FP+TN+FN}$$

(5) $$Precision=\frac{TP}{TP+FP}$$

(6) $$Precision=\frac{TP}{TP+FN}$$

(7) $$F1\text{-}Score=2\times \left(Precision\times Recall\right)\;/\;\left(Precision+Recall\right)$$

### 4.3. Performance Evaluation Based on the Proposed Network

This section provides a brief comparison of the proposed network against various competitive techniques in terms of quantitative and qualitative analysis over two challenging datasets, as discussed in the subsequent subsection.

#### 4.3.1. Quantitative Comparison of the Proposed Network with Other Techniques

This subsection presents a detailed comparison of the proposed network and various competitive techniques over two benchmarks using distinct evaluation indicators such as accuracy, precision, recall, and F1 score, as tabulated in Table 3. In the experimental analysis, the proposed dual stream with soft-attention network attained superior performance over custom dataset, representing 90.89%, 91.11%, 90.84%, and 90.85% for accuracy, precision, recall, and F1 score respectively. In addition, DenseNet121 is considered a second-best model for our custom dataset, which obtained 89.81% accuracy, 91.1% precision, 90.47% recall, and 90.53% F1 score respectively. The internal architecture of our dual-stream network utilizes a blend of residual and inception blocks, including an attention mechanism to emphasize significant characteristics. This strategy enables the model to effectively capture complex patterns, especially when dealing with challenging input data, leading to enhanced accuracy in classification. Unlike several competitive techniques that struggle with precision and recall, our proposed network demonstrates a balanced performance between accuracy and the capacity to accurately identify pertinent instances of plant species.

To further examine the effectiveness and scalability of the proposed network, we provided a detailed assessment of the network with benchmark Flower299 dataset using different evaluation indicators for instance accuracy, precision, recall, and F1 score. As given in Table 3, the proposed network demonstrates outstanding performance for accuracy, precision, recall, and F1 score, showcasing 88.87%, 88.98%, 88.64% and 88.92%, respectively. Further, the DensNet169 achieved promising performance for accuracy, precision, recall, and F1 score, which is 87.43%, 87.3%, 86.94%, and 86.95%, respectively. In addition, the DenseNet169 is considered the second-best network among others over Flower299 dataset. In short, the detailed experimental analysis of the proposed network over custom dataset, and a publicly available dataset, justified the robustness of the proposed network while handling the intricacies of the plant classification problem, positions it as a possible paradigm for other image classification domains.

#### 4.3.2. Qualitative Analysis in Terms of Visualized Result

**Figure 4:**
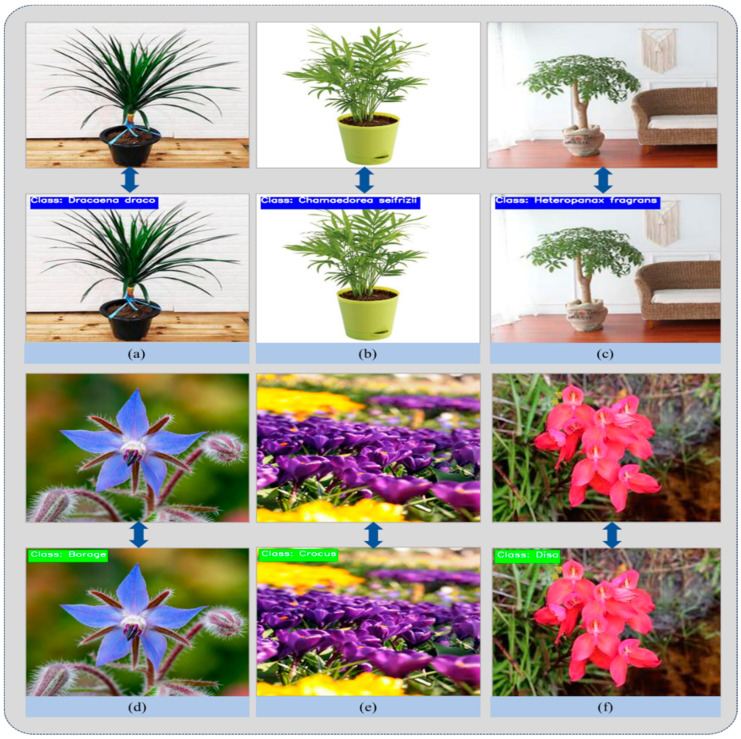
Demonstrates the classification performance of our proposed model on testing samples, where (**a**–**c**) shows samples from our custom dataset, while (**d**–**f**) shows samples from the Flower299 dataset.

We conducted an investigation into the qualitative analysis of a suggested model that combines inception and residual blocks, followed by a soft-attention mechanism. This model shows substantial improvements in plant species categorization. The model efficiently extracts complete features from plant images by using the capabilities of inception blocks for collecting multi-scale characteristics and residual blocks for fast deep network training. Incorporating dilated convolutional layers significantly improves the model’s capacity to collect contextual information across different scales. The soft-attention mechanism is essential for directing attention toward the most relevant regions of the image, hence enhancing the model’s capacity to differentiate between similar plant species. The use of this attention mechanism enables the model to dynamically emphasize important characteristics, resulting in enhanced precision and resilience in categorization. The qualitative findings are visualized in Figure 4, demonstrating that the suggested architecture achieves superior classification accuracy and improved generalization across various plant species. This highlights its usefulness and promise for practical applications in plant biology and agriculture.

#### 4.3.3. Discussion

This section demonstrates the experimental findings and discussion of our proposed dual-stream architecture enhanced by a soft-attention mechanism for plant species classification. With the addition of a soft-attention mechanism, our model improves the precision and comprehensibility of species classification. The experiments are performed on two different datasets, which have a wide range of plant species. We have conducted thorough experimentation using evaluation metrics like accuracy, precision, recall, and F1-score to assess the model performance. Furthermore, the discussion analyzes the advantages and possible drawbacks of the model, which provides a comprehensive assessment of the model’s performance for future improvement in the field of plant species classification.

The incorporation of residual and inception blocks provides an optimal combination of feature-extraction capabilities. Residual block, by using skip connections and the capacity to learn residual mappings, enhances the seamless transmission of information throughout the network, addressing the issue of vanishing gradients and allowing the model to effectively capture complex characteristics. The inception block enhances the receptive field by using filters of different sizes simultaneously, enabling the model to efficiently collect information at several scales. Furthermore, the integration of a soft-attention mechanism after the fusion of features from residual and inception blocks provides an additional level of flexibility to the model. The soft-attention mechanism selectively provides weights to certain regions of the feature maps, prioritizing areas that are essential for precise classification. The model’s flexibility improves its capacity to focus on key facts while excluding unnecessary or duplicate information. Figure 4 demonstrates the classification performance of our proposed model on testing samples.

The effectiveness of the proposed model is credited to the integration of these architectural components, which are skilled at capturing complex and unique features from the input data. The model accomplishes a high Accuracy, Precision, Recall, and F1-score by merging the advantages of residual and inception blocks, as well as including a soft-attention mechanism. This guarantees accurate and effective classification and comprehensive coverage of all plant species attributes. The model’s ability to extract complex features and allocate attention in a nuanced manner allows it to identify subtle patterns in images, resulting in improved performance compared to other benchmark models.

### 4.4. Ablation Study

We performed a detailed ablation study to select the most optimal model for plant species classification. Initially, we use the simple CNN model by utilizing the residual and inception blocks for classification. The CNN architecture is well-suited for plant species classification tasks. The process begins with a convolutional layer that has 64 filters and a 7 × 7 kernel size by providing the input structure of 224 × 224 × 3. The features are then downsampled using batch normalization and max pooling. Next, the network is made up of inception and residual blocks, which are both renowned for their ability to capture hierarchical characteristics. Shortcut connections are included to reduce the occurrence of vanishing gradient issues and promote a smoother gradient flow during training. To extract various features, the inception block uses max pooling and parallel convolutional procedures with varied kernel sizes. Average pooling is used after each block to flatten the representation of features for dense layers and minimize spatial dimensions. Densely connected nodes make up the fully connected layers, which lower dimensionality while retaining higher-level abstractions. To reduce overfitting, dropout regularization is used. Softmax activation is used in the last layer to provide probabilities for classes, where it varies from dataset to dataset, making it appropriate for multi-class plant species classification problems. To accomplish efficient feature extraction and classification, this kind of model architecture combines conventional convolutional layers with novel residual and inception blocks to obtain and train hierarchical representations from images.

Second architecture designed for plant species classification is implemented where network begins with an input layer that has dimensions of 224 × 224 × 3, which represent the width, height, and three RGB channels of the image. Next, it employs dilated convolutional layers with a 7 × 7 kernel and a stride of 2, followed by batch normalization and max-pool operations to effectively extract low-level features and minimize the data spatially. Residual block is incorporated into the model where each consists of two 3 × 3 convolutional layers that are equipped with skip connections and batch normalization. Then, inception block with their parallel dilated convolutional branches (1 × 1, 3 × 3, and 5 × 5 convolutions) and max-pooling functions allow for the extraction of features at different scales, which improves the network’s comprehension of complex images. The inception block is followed by an attention mechanism. To compute attention weights, this approach first uses dense layers, then global average pooling. To improve the network’s attention on critical image regions for classification, these weights are rearranged and multiplied elementwise with the feature maps. The goal is to amplify informative regions and decrease less relevant parts. The approach progresses from condensing feature maps by average pooling to flattening and completely correlated layers, which accomplish high-level feature abstraction. In the end, dropout regularization and Softmax layers are used for multi-class categorization over several classes, which completes the network’s architecture.

The third architecture consists of two separate paths that handle the input data through different phases of pooling and specific convolutional operations to enable different feature-extraction techniques. The initial step is to define the input tensors that represent the two parallel streams, residual block, and inception block. Each stream travels through a series of pooling and dilated convolutional layers that are specific to the block type it is assigned. The first stream, in particular, integrates residual block, which are distinguished by their capacity to maintain gradient flow and enable feature learning via shortcut connections. Parallelly, the second stream utilizes the inception block to obtain complicated features by using various receptive fields via parallel convolutional routes. The concatenate layer concatenates the features from both streams, whereas the concatenated feature representations from both streams enable the combination of complementary and varied features. This layer is followed by dense layers and output layers for the classification of plant species. The advantage of this architecture is that it utilizes the inception and residual block for enhancing the feature representation from two different streams where the gradient degradation is tackled by the residual block and feature hierarchies are captured through various receptive fields that is performed by the inception block. This approach attempts to learn more complimentary feature representation by combining the features from both parallel streams. This approach has the potential to improve the learning strategies in various computer vision applications and classification tasks by collecting a variety of features from different streams.

### 4.5. Drawbacks Associated with the Proposed Network

While the proposed network exhibited an optimal performance using two challenging datasets, as evident in the detailed experimental results, the model is associated with various drawbacks, as structured in the following points:

- The proposed network comprises complex architecture, employing multiple streams followed by an attention mechanism. This complexity enhances its ability to capture complex patterns within input data, leading to improved overall performance. However, it can be challenging to manage and optimize such complex architecture, particularly during training.
- Another limitation associated with the proposed model is high computational complexity, which can be challenging when deploying the model over edge devices for real-time decision-making. This limitation needs to be carefully considered when planning for deployment in resource-constrained environments.
- The complexity of the network may lead to the risk of overfitting, particularly when training the network on limited or unbalanced datasets. Mitigating this risk may require additional regularization techniques or a larger and more diverse training dataset.

## 5. Conclusions and Future Directions

Our proposed dual-stream framework, followed by a soft-attention mechanism, demonstrates a strong and innovative system for classifying plant species where we utilized the residual block in the first stream and the inception block in the second stream. The utilization of dilated convolutional layers in both streams improves the model’s receptive field, making it capable of gathering features from a broader region in the images. The complex feature-extraction capacity is essential for identifying complex patterns and characteristics seen in plant species images. The combination of residual and inception blocks exploits the advantages of both architectures, enhancing the variety of features and producing detailed representations. Moreover, the residual block facilitates the mode of handling the vanishing gradient problem, while the inception block enhances the receptive field for extracting more prominent features from the data. The use of dilated convolutions also enhances the model’s capacity to extract characteristics at various sizes without substantially increasing computational complexity. Our proposed architecture has a soft-attention mechanism after the concatenation of feature maps from both streams, which is a distinctive characteristic of this architecture. The use of this soft attention enables the model to flexibly concentrate on more appropriate regions, hence enhancing its capability to extract more features. The attention process is essential for identifying small differences among different plant species, which contributes to the high accuracy in classification. Our suggested architecture has shown exceptional performance in plant species classification tasks via rigorous testing and assessment. Our model outperforms other baseline models in plant species classification for botanical applications because of the meticulous incorporation of many elements like the utilization of dilated convolutions in residual and inception blocks and the soft-attention mechanism. Our proposed model for plant science demonstrates the effectiveness of mixing distinct architectural aspects to enhance accuracy and generalization in recognizing plant species.

When considering the future trajectory of our suggested design, various intriguing paths might be explored and improved upon. Initially, conducting more research on enhancing hyperparameters and refining the model architecture might result in even more remarkable improvements in performance. Investigating the interpretability and explainability features of the model’s predictions might improve the model’s usefulness in real-world situations. Moreover, the expansion of the dual-stream design to include multi-modal inputs, such as extra spectral or temporal data, shows potential for thorough plant species classification. By integrating a variety of data sources, a comprehensive understanding of plant ecosystems may be achieved, allowing the model to accurately capture subtle patterns and changes. Gaining insight into the primary factors that influence a certain categorization choice may enhance the confidence and acceptance of the model in agricultural and ecological research. As technology progresses, the suggested framework in edge computing settings, such as agricultural drones or field-based sensors, has the potential to revolutionize real-time plant species identification. Creating lighter versions of the model and incorporating it onto edge devices might provide on-site, in-field applications with minimum delay.

## Figures and Tables

**Table 1:** Representation of the summary of previous techniques used for plant classification.

Ref	Datasets	Classes	Models	Accuracy	
[7]	Custom dataset	30	DNN	91.17%	
		30	CNN	95.58%	
[8]	Custom dataset	15	VGG16	91.50%	
		15	VGG19	92.40%	
		15	Inception-ResNetV2	89.60%	
[11]	LeafSnap	180	ResNet	93.09%	
[29]	PlantVillage	38	AlexNet	99.27%	
		38	GoogLeNet	99.34%	
[30]	Six different datasets	22	CNN	86.20%	
[32]	Vietnam Plant (VNP-200)	200	CNN-PC	96.42%	
[33]	QuangNamForestPlant	710	Masked Autoencoder	94%	
[34]	LeafSnap	184	LeafNet	86.30%	
		60	LeafNet	95.80%	
		32	LeafNet	97.90%	
[35]	Malayakew	44	Deep CNN (D1) MLP	97.70%	
		44	Deep CNN (D1) SVM (linear)	98.10%	
[37]	LifeCLEF 2015	30	3SN	84.20%	
[39]	AyurLeaf	40	AlexNet	94.87%	
		40	Ayurleaf CNN	95.06%	
[40]	Custom dataset	20	MobileNet	98.50%	
[41]	Flavia	32	Ten-layer CNN model	87.92%	
[43]	Custom dataset	3	AlexNet	97.80%	

**Table 2:** Represents the summary of both datasets.

Dataset	Total Images	Training	Testing	No. of Classes
Flower299	115,944	92,755	23,188	299
Proposed Dataset	9364	7492	1872	48

**Table 3:** Comparative analysis of our proposed model against various competitive networks over custom dataset and Flower299.

No.	Model	Custom Dataset				Flower299 Dataset			
		Acc.	Prec.	Rec.	F1	Acc.	Prec.	Rec.	F1
1	DenseNet121	89.81	91.1	90.47	90.53	84.69	84.41	84.15	84.16
2	DensNet169	85.25	85.73	84.72	84.76	87.43	87.3	86.94	86.95
3	EffecientNetB0	78.13	78.9	77.03	78.10	78.67	78.1	78.88	78.65
4	InceptionResNet	76.06	78.84	74.95	75.04	83.55	84.45	83.1	83.67
5	InceptionV3	84.5	85.28	83.77	83.77	84.52	84.36	84.03	84.05
6	ResNet50	88.35	88.9	88.13	88.23	84.76	84.53	84.26	84.26
7	MobileNetV2	85.84	86.57	85.22	85.37	85.15	84.89	84.65	84.64
8	VGG16	88.9	90.61	90.1	90.19	84.34	84.16	83.84	83.87
9	VGG19	88.3	88.99	88.29	88.30	84.48	84.29	83.97	84.01
10	Inc-Res	85.84	86.57	85.22	85.37	82.48	82.78	82.2	82.75
11	Inc-Res SAM	85.3	85.8	84.7	84.81	83.15	83.6	83.02	83.45
12	Dual Str. Inc-Res	87.09	87.39	87.29	87.18	84.65	85.2	83.9	84.78
13	Proposed Model	90.89	91.11	90.84	90.85	88.87	88.98	88.67	88.92

## Data Availability

Data are contained within the article.
